# Genome-wide identification of clusters of predicted microRNA binding sites as microRNA sponge candidates

**DOI:** 10.1371/journal.pone.0202369

**Published:** 2018-08-24

**Authors:** Xiaoyong Pan, Anne Wenzel, Lars Juhl Jensen, Jan Gorodkin

**Affiliations:** 1 Center for non-coding RNA in Technology and Health, Department of Veterinary and Animal Sciences, University of Copenhagen, Frederiksberg, Denmark; 2 Disease Systems Biology Program, Novo Nordisk Foundation Center for Protein Research, University of Copenhagen, Copenhagen, Denmark; Ben-Gurion University, ISRAEL

## Abstract

The number of discovered natural miRNA sponges in plants, viruses, and mammals is increasing steadily. Some sponges like ciRS-7 for miR-7 contain multiple nearby miRNA binding sites. We hypothesize that such clusters of miRNA binding sites on the genome can function together as a sponge. No systematic effort has been made in search for clusters of miRNA targets. Here, we, to our knowledge, make the first genome-wide target site predictions for clusters of mature human miRNAs. For each miRNA, we predict the target sites on a genome-wide scale, build a graph with edge weights based on the pairwise distances between sites, and apply Markov clustering to identify genomic regions with high binding site density. Significant clusters are then extracted based on cluster size difference between real and shuffled genomes preserving local properties such as the GC content. We then use conservation and binding energy to filter a final set of miRNA target site clusters or sponge candidates. Our pipeline predicts 3673 sponge candidates for 1250 miRNAs, including the experimentally verified miR-7 sponge ciRS-7. In addition, we point explicitly to 19 high-confidence candidates overlapping annotated genomic sequence. The full list of candidates is freely available at http://rth.dk/resources/mirnasponge, where detailed properties for individual candidates can be explored, such as alignment details, conservation, accessibility and target profiles, which facilitates selection of sponge candidates for further context specific analysis.

## Introduction

MicroRNAs (miRNAs) are a class of endogenous small non-coding RNAs of about 20 nucleotides in length, which play crucial roles in transcriptional and post-transcriptional control of gene expression through interacting with other RNAs [1–4]. To date, more than 2000 human mature miRNAs (miRBase v20) [5] have been discovered. These mature miRNAs are formed from pre-miRNAs, which are processed by DICER in the cytoplasm [6]. They are estimated to regulate more than 60% of all human protein-coding genes (PCGs) [7] and have been implicated in many human diseases [8, 9]. With more and more miRNAs being discovered, identifying their functions is becoming increasingly important for understanding the molecular mechanisms of diseases [10].

The miRNAs can themselves be regulated by so-called miRNA sponges, which are RNAs with many miRNA binding sites that compete with the target sites for binding of one or more miRNAs of interest. Denzler et al. analyzed the relationship between sponge activity and number of binding sites and found that more binding sites enhance miRNA sponge effect on releasing mRNA target repression regulated by that miRNA [11]. Artificial miRNA sponges have been used to generate loss-of-function phenotypes for miRNAs in cell culture [12] and to discover miRNA functions *in vivo* [13]. It has advantages over genetic knock-outs and antisense oligonucleotide inhibitors by being cheaper and less time consuming [14]. They are also of therapeutic interest [15, 16].

Natural miRNA sponges with many miRNA binding sites separated by linker regions also exist [17] (S1 Table). They have also been called competing endogenous RNA (ceRNA), and the ceRNA hypothesis suggests that RNAs regulate each other by competing for shared miRNAs [18]. Recently, a circular RNA (circRNA) with more than 70 binding sites was shown to function as a sponge for miR-7 [19, 20]. This natural sponge, named ciRS-7 and CDR1as, has been implicated in cancer-related pathways [21]. A circRNA derived from the gene encoding zinc finger protein 91 (circRNA-ZNF91) with 24 miR-23 binding sites has similarly been identified as a possible miRNA sponge [22]. However, some studies consistently mention that only few circRNAs can function as miRNA sponges [22, 23]. Other types of transcripts can also serve as natural miRNA sponges, such as the pseudogene PTENP1 [24] and the long non-coding RNAs (lncRNAs) H19 [25] and lincRNA-RoR [26]. It has been estimated that there are thousands of RNA transcripts functioning as potential miRNA natural sponges [27], but despite increasing evidence for the ceRNA hypothesis, it still attracts some skepticism [28]. Although there exist several compilations of putative ceRNAs derived from predicted miRNA target sites, CLIP-Seq data, or both [29–31], reviewed in [32], none of the studies to date have systematically analyzed the genome for clusters of miRNA binding sites.

In an attempt to shed more light on this, we here analyze the complete human genome for clusters of predicted miRNA target sites, which may represent natural miRNA sponges. To this end, we identify statistically significant clusters by comparing the numbers of binding sites in the clusters obtained from the real genome and from shuffled genomes, retaining the local sequence composition. We further filter the resulting clusters based on evolutionary conservation and binding energies. With this approach we rediscover one known miRNA sponge ciRS-7 for miR-7 and identify 3672 novel sponge candidates.

## Materials and methods

### Data sources

The repeat-masked human genome sequence (hg19) was downloaded from the UCSC Genome Browser database [33]. All 2578 human mature miRNAs were extracted from miRBase v20 [5]. GENCODE v19 [34] and circBase [35] were used to annotate sponge candidates, which cover protein-coding genes, lincRNAs, circRNAs, antisense (overlaps a protein-coding locus on the opposite strand), pseudogene, and processed_transcript (a transcript without an open reading frame). To get information about binding site conservation, phyloP (phylogenetic P-values) scores [36] were downloaded from ftp://hgdownload.cse.ucsc.edu/goldenPath/hg19/phyloP46way/.

### Pipeline for clustering of miRNA target sites

**Fig 1:**
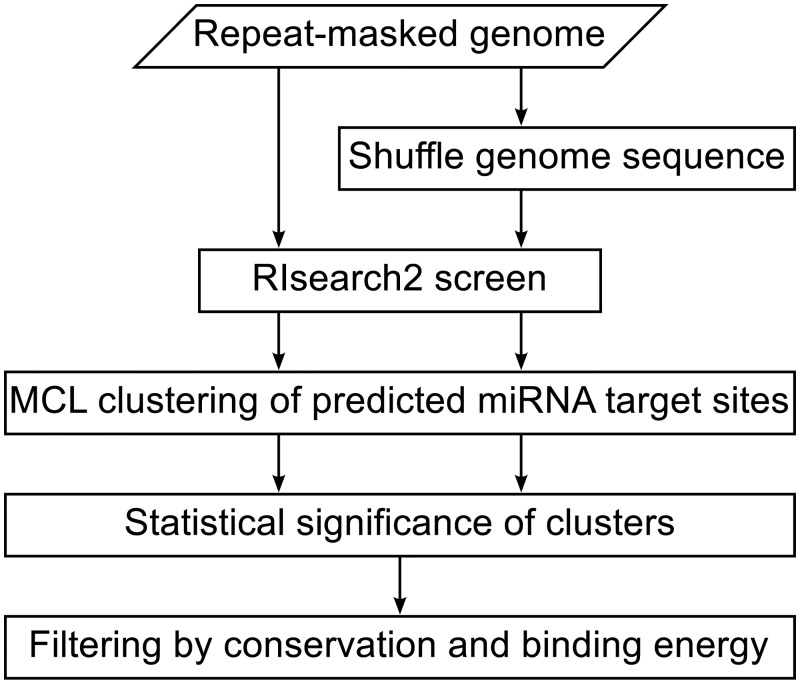
Flowchart of the analysis pipeline. For each mature miRNA in miRBase v20, we ran RIsearch2 against both the real repeat-masked genome and a shuffled version to predict binding sites. We then used the Markov Cluster (MCL) algorithm to identify genomic clusters of binding sites and identified statistically significant clusters by comparing the results for the real and shuffled genomes. Finally, the significant clusters were further filtered by conservation and binding energy.

To identify genomic clusters of predicted miRNA target sites for a given miRNA, we have developed a pipeline as outlined in Fig 1.

#### RIsearch2 screen for miRNA target sites

The computational screen on hg19 for target sites of the miRBase miRNAs was performed with a preliminary version of RIsearch2 [37]. It is a seed-and-extend approach to predict RNA–RNA interactions, applying suffix arrays in the first stage to locate initial seed matches (allowing for G–U wobble matches) and using dynamic programming (DP) to extend those matches with the simplified energy model as introduced in RIsearch [38]. The seed was specified to require a stretch of six consecutive bases within the first eight bases of the miRNA sequence to be paired. The window for DP extension was set to always include the entire remaining query sequence outside the seed, and the same number of nucleotides extended by five from the target. This parameter from the preliminary version of RIsearch2 used in here has been replaced with a maximum extension length in the released version. The default value of 20 nt should yield comparable results. The maximum hybridization energy was set to −10 kcal/mol. Overlapping target sites were merged in post-processing.

#### Markov clustering of predicted miRNA target sites

To identify genomic regions with a high density of predicted binding sites for a given miRNA, we used the Markov Cluster (MCL) algorithm [39], which does not need specify the number of clusters in advance. In our pipeline, we run clustering for individual miRNAs, and each miRNA has different number of clusters.

To this end, we represented the predicted binding sites for each miRNA as a weighted network, in which the weight of the edge between two sites on the same strand of the same chromosome is defined based on their nucleotide distance (*x*) as follows:

$$sim\left(x\right)=\{\begin{array}{cc} C-x & \;\text{if}\;x<C\\ 0 & \;\text{if}\;x\geq C \end{array}$$ (1)

where the constant *C* = 1000 determines over which distance the weights decay. The value of *C* was chosen to allow identification of large clusters while limiting the computational cost. Clusters within this weighted network were then identified using the MCL algorithm with a range of different values for the inflation factor parameter, which influences the size and number of clusters.

#### Shuffling of genome sequence

We evaluate the statistical significance of the identified clusters by creating a background model from randomized genome sequence and repeating the RIsearch2 and MCL steps described above. To preserve the local dinucleotide content, the non-masked sequence segments of the human genome are shuffled by uShuffle [40] in non-overlapping windows of 120 nt, the typical size of structured RNA [41, 42].

#### Statistical significance of clusters

For each miRNA, we estimate a cutoff on the number of predicted target sites in a cluster that is required for statistical significance. This is done by fitting the size distribution of the top-10% largest clusters obtained for the miRNA in question on the randomized genome, assuming an exponential tail.

$${\text{log}}_{10}\left(y\right)=a\cdot x+b$$ (2)

where *x* is the cluster size and *y* is the number of clusters of a given size. Based on this fit, we extrapolate the largest cluster one would expect to observe in 1000 randomizations.

$$x_{cutoff}=\frac{{\text{log}}_{10}\left(1/1000\right)-b}{a}$$ (3)

Only clusters larger than or equal to this cutoff are considered statistically significant. We used this approximation because it would take prohibitively long time to run MCL clustering on the RIsearch2 output for thousands of randomized genomes for every miRNA.

#### Filtering by conservation and binding energy

To further improve the quality of the predictions, we apply two additional filtering criteria to the statistically significant clusters. First, we extract the evolutionarily conserved subset of target site predictions on the real genome, by requiring that the miRNA seed site has at least five continuous nucleotides with a phyloP score greater than 0.3 [20]. For individual miRNAs, clusters with the percent of conserved sites smaller than 2 times as one would expect to observe in whole genome (the number of conserved binding sites vs the number of all binding sites for this miRNA) are excluded. Second, we filter out statistically significant clusters that are caused by repetitive sequences not masked in the downloaded genome, because RepeatMasker and Tandem Repeats Finder by default only mask simple repeats with a unit length up to 12 and curated repeats from Repbase [43, 44]. Instead of rerunning RepeatMasker with different parameters, we use the binding energies already calculated by RIsearch2 to eliminate clusters for which many sites have the exact same predicted binding energy. To this end, we calculate the normalized Shannon entropy of the binding site energies in each cluster as follows:

$$entropy=-\sum_{i=1}^{m}p_{i}\cdot {\text{log}}_{2}\left(p_{i}\right)/{\text{log}}_{2}\left(n\right)$$ (4)

where *m* is the number of different binding energy values found in the given cluster, *p*_*i*_ is the relative frequency of a particular energy in there, *n* is the number of binding sites in the cluster. We used an entropy threshold of 0.6 to filter out clusters that have many sites caused by repetitive sequences.

### Characterization of sponge candidates

To characterize the sponge candidates within the web interface, we annotate them with overlapping genes based on their coordinates and strand using genes from GENCODE v19 and circBase. Thus we create a reference annotation in which GENCODE annotation is primary and all annotation from circBase not overlapping that of GENCODE is included as additional annotation. We further calculate a number of properties for each sponge candidate, which are described in the following sections.

#### SNP density ratio

CircRNAs have significant lower SNP density at miRNA seed sites than in their flanking regions and other sites, suggesting selective pressure to maintain those binding sites [45]. We calculated the SNP density ratio (SDR) between miRNA seed sites and the remaining sequence. For every binding site within a sponge, the seven nucleotides base-pairing with the miRNA seed region (positions 2–8) are defined as miRNA seed site. The gap region between every two seed sites is defined as flanking region, hence also including regions base-pairing with the miRNA outside the seed. The SDR is calculated as ratio between the SNP density in miRNA seed sites and flanking region, shown in Eq (5). SNP data was downloaded from Ensembl 75 [46].

$$\begin{array}{c} \begin{array}{c} SNPdensity=\frac{\#\;of\;SNPs}{\#\;of\;nucleotides}\\ SDR=\frac{SNPdensity\;in\;seed\;sites}{SNPdensity\;in\;flanking\;region} \end{array} \end{array}$$ (5)

#### Fraction of binding sites within exons

It is furthermore important whether the predicted binding sites are intronic or exonic [22]. We thus calculate the fraction of binding sites in the sponge candidate that fall within exons (FBSE) based on GENCODE v19 as follows:

$$FBSE=\frac{\#\;of\;binding\;sites\;within\;exon}{\#\;of\;binding\;sites\;within\;sponge}$$ (6)

#### Energy and fraction of paired nucleotides for binding sites

For each sponge candidate, we plot the predicted binding site energies and compare them to the distribution of binding site energies for known targets of the same miRNA. We calculated the latter by running RIsearch2 on the 3’ UTRs from Ensembl 75 of the known and experimentally identified miRNA targets in the RAIN database [47]. We similarly plot the fraction of paired nucleotides for the binding sites, which is defined as the number of base-pairings between the binding site and the miRNA divided by the length of the mature miRNA.

#### Visualization of sponge candidates in genome browser

To allow for visualization of sponge candidates in the UCSC browser [33], we display tracks with the position, binding energy, conservation, and accessibility of each binding site. The accessibility track contains the probability of each nucleotide being unpaired within the internal structure of the transcript, estimated using RNAplfold [48] with a maximum base pair span of 120 nt and window size 170 nt.

## Results

### Clusters of predicted miRNA binding sites

The first step in searching for clusters of predicted miRNA binding sites is to predict the binding sites themselves. To do that several tools can potentially be employed; however, doing a large-scale screen on the complete human genome requires speed. We primarily focus on RIsearch2 as it was benchmarked against miRNA tools and found to be substantially faster than other RNA–RNA interaction prediction tools [37]. We justify this choice by comparing the RIsearch2-based screen to screens based on a GUUGle search (using a minimum match size of six nucleotides) [49] and a relaxed BLAST search (E-value < 10 000) [50]. For all three tools we subsequently employ the MCL clustering algorithm (with the default inflation factor of 2.0) to identify clusters in the genome with a high density of predicted binding sites for a given miRNA.

We used RIsearch2 to search the 2578 mature miRNAs from miRBase (v20) against the human genome (hg19) and its shuffled counterpart. For each miRNA, we subsequently used MCL to identify clusters of predicted miRNA binding sites and compared the number of clusters obtained on the real and shuffled genomes as function of cluster size (S2 Table). Whereas we were able to perform this analysis for all miRNAs using RIsearch2, this was not feasible for the GUUGle screen due to the large number of predicted miRNA binding sites.

**Fig 2:**
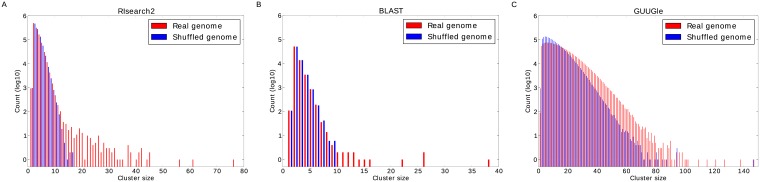
Cluster size distribution for predicted miR-7 binding sites. The plots show the size distributions of the clusters obtained for the real and shuffled genomes when running MCL clustering with an inflation factor of 2.0 on the miR-7 binding sites predicted by (A) RIsearch2, (B) BLAST, and (C) GUUGle.

We thus instead, as an example, compare the results from the three methods for miR-7 (Fig 2). For the RIsearch2 screen (Fig 2A) we obtain far more large clusters on the real genome than on the shuffled one. For the BLAST search (Fig 2B) we see a similar but much weaker trend with fewer clusters both on the real and shuffled genome. A possible explanation is that BLAST does not allow G–U base pairing and thus predicts much fewer binding sites. In contrast, GUUGle predicts many more binding sites resulting in more and larger clusters (Fig 2C). However, we observe only very small differences between the real and the shuffled genomes. It should be noted, that GUUGle is intended to be used as a prefilter for more sophisticated but computationally expensive methods. However, as RIsearch2 evaluates the thermodynamic strength of the predicted binding sites and is nonetheless faster [37], we opted to use RIsearch2.

To further illustrate the predictive power of RIsearch2, we compare its predicted miR-7 binding sites to those of TargetScan [51] for the natural sponge ciRS-7 [19]. Both RIsearch2 and TargetScan predict 73 binding sites, of which 72 are in common. The one binding site found by TargetScan, but not by RIsearch2, has a predicted binding energy of −9.87 kcal/mol, which is only slightly above the energy cutoff (−10 kcal/mol). By contrast, the BLAST screen predicts only seven binding sites. While giving comparable results to TargetScan, RIsearch2 runs approximately four times faster.

**Fig 3:**
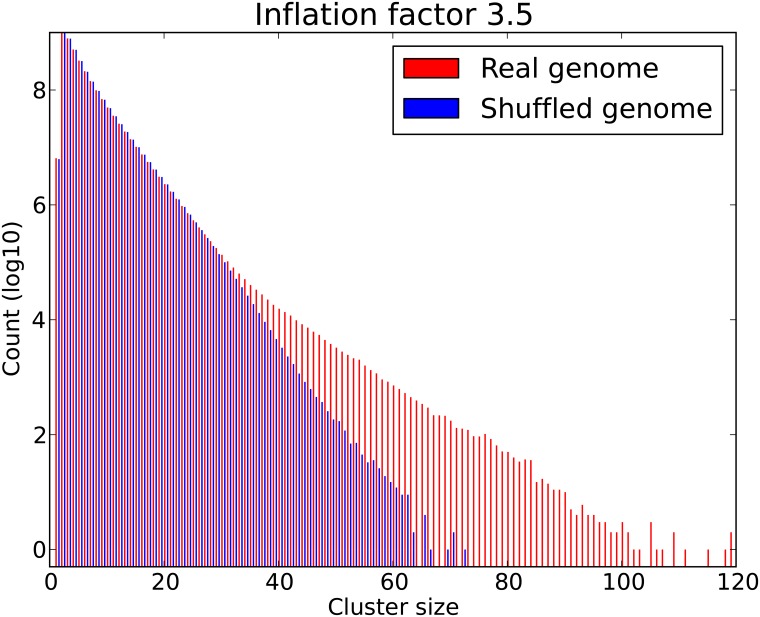
Overall cluster size distribution of miRNA binding sites predicted by RIsearch2. The plot shows the size distributions obtained for real and shuffled genomes when pooling the results for 2578 mature human miRNAs. For each miRNA, we used RIsearch2 to predict binding sites and clustered them using MCL with inflation factor 3.5.

Whereas RIsearch2 is our preferred miRNA binding sites predictor, we can further improve the results by changing the main parameter in MCL, the inflation factor, which affects the granularity of the clusters. To this end, we ran our pipeline with inflation factors 1.5, 2.0, 2.5, 3.0, 3.5 and 4.0 for each miRNA on both the real and shuffled genomes. These runs took roughly three months to compute on a cluster with 16 nodes, each equipped with two Intel Xeon E5-2650 processors, having a total of 256 cores. For each inflation factor, we pooled the resulting clusters for all miRNAs and plotted the cluster size distributions (S1 Fig). As anticipated, higher inflation factors led to smaller clusters. Based on visual inspection of the differences between real and shuffled genomes, we decided to use an inflation factor of 3.5 (Fig 3).

The more predicted miRNA binding sites a cluster contains, the less likely it is to occur in a shuffled genome. We can thus estimate the statistical significance of a cluster based on its size. Since different miRNAs give rise to different cluster size distributions on shuffled genomes (S2 Fig), significance analysis is done separately for each miRNA. In principle, one could estimate the false discovery rate corresponding to a given cutoff on cluster size based on the empirical distribution obtained from thousands of shuffled genomes. However, as this is not computationally feasible, we instead fit the tail of the distribution obtained from a single shuffled genome to find a cluster size cutoff for each miRNA. For example, although the largest miR-7 cluster found in the shuffled genome contains 15 predicted binding sites, we extrapolate that a miR-7 cluster must contain at least 20 binding sites to have less than 0.1% probability of appearing by chance alone. We refer to this as the empirical p-value.

As a control we also checked if we obtained the same number of predicted miRNA binding sites in the real and shuffled genome. These numbers (at the −10 kcal/mol cutoff) are 3 430 423 948 and 3 386 114 664, with a ratio of 1.013 constituting no bias in the volume of binding sites in the two genomes.

### Sponge candidates

Using the pipeline shown in Fig 1 to the second last step, we obtained a total of 71 106 statistically significant (*P* < 0.001) clusters of binding sites for 2543 mature miRNAs (for 35 mature miRNAs we obtained no significant clusters). To identify the ones most likely to be of biological relevance, we employ the last step by filtering for conservation and binding energy (see Methods for details). This reduced the clusters to 3673 sponge candidates for 1250 miRNAs, which can all be viewed and downloaded via our web resource (http://rth.dk/resources/mirnasponge).

**Fig 4:**
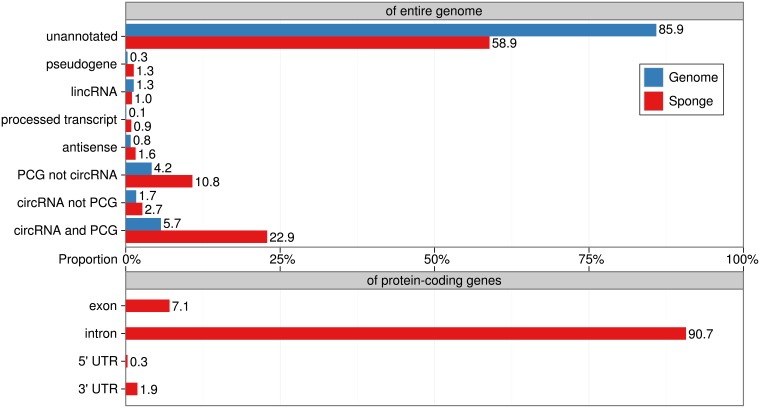
Genomic context of the sponge candidates. The upper bar chart shows the percentage for the different types of transcripts in the genome based on GENCODE and circBase, and their percentage within our sponge predictions are calculated after we assign annotations to the predicted sponge candidates. For each type of transcript, we calculate the percentage of their nucleotides under whole genome and annotated sponges. Then we can evaluate the enrichment via comparing the percent between sponges and whole genome. There is big overlap between PCGs and circRNAs, so we further divide them into “PCG not circRNA”, “circRNA not PCG” and “circRNA and PCG”. They refer to PCGs not overlapping with circRNAs, circRNAs not overlapping with PCGs, and PCGs overlapping with circRNAs, respectively. The lower bar chart shows the percentage of nucleotides located in intron, exon, 3’ UTR, and 5’ UTR for all annotated PCG sponge candidates. All percentages are calculated based on the number of nucleotides, excluding masked repeats, and are strand-sensitive.

To annotate the sponge candidates with presumed genes of origin, we compared the genome coordinates of each sponge to annotated circRNAs from circBase and other genes from GENCODE. We annotated a sponge candidate to a gene if the larger of these two genomic regions covered at least 50% of the smaller one. Given this criterion, the majority of our candidates (2162 out of 3673) fall in unannotated genomic regions, which is not surprising considering that 85.9% of the genome is not covered by either GENCODE or circBase (Fig 4, upper panel). Most notably, the predicted sponges are enriched for overlaps with circRNAs and protein-coding genes. In particular the circRNA sponges overlapping with PCGs have four times (22.9%) as many sponges as what one would expect by chance (5.7%) from the genomic annotation. The overlaps of sponge candidates with specific parts of protein-coding genes (intron, exon, 3’ UTR, and 5’ UTR) are shown in Fig 4 (lower panel).

To identify a subset of sponge candidates of particular interest, we first select the 768 sponge candidates that have at least 10 predicted binding sites more than what is required for the significance cutoff. Of these we focus on the subset that could be annotated with known genes and further require that at least 9% of the predicted binding sites reside within exons (FBSE > 0.09, which is the mean value of FBSEs for sponge candidates annotated with known genes). These sponge candidates are listed in Table 1 and include the known natural sponge ciRS-7.

**Table 1:** Filtered miRNA sponge candidates. The table provides an overview of miRNA sponge candidates that have at least 10 binding sites more than what is required for statistical significance, can be annotated with known genes, and have more than 9% of predicted miRNA binding sites within exons. The column *cluster size* lists the number of binding sites in the given cluster for real genome and the cluster size cutoff for statistical significance obtained from shuffling. The sponge candidates are sorted based on the difference between these two cluster sizes.

miRNA	Cluster size real / cutoff		Genomic coordinate chromosome: range (strand)	Annotation
hsa-miR-7-5p	76	20	chrX: 139 865 250–139 866 947 (+)	circRNA: ciRS-7
hsa-miR-4310	54	26	chr22: 50 671 491–50 673 762 (−)	protein_coding: TUBGCP6
hsa-miR-376b-5p	38	18	chr15: 101 093 988–101 095 732 (−)	pseudogene: PRKXP1
hsa-miR-766-5p	70	51	chrX: 139 865 341–139 867 009 (+)	circRNA: ciRS-7
hsa-miR-4295	35	17	chr16: 690 762–691 826 (−)	pseudogene: AL022341.1
hsa-miR-4729	44	27	chr16: 90 060 979–90 062 561 (+)	circRNA: hsa_circ_0041137
hsa-miR-190b	38	21	chr17: 412 328–413 728 (+)	antisense: RP5-1029F21.3
hsa-miR-93-3p	61	46	chr16: 90 060 884–90 062 555 (+)	circRNA: hsa_circ_0041137
hsa-miR-8077	52	38	chr17: 80 211 257–80 213 687 (−)	circRNA: hsa_circ_0046395
hsa-miR-545-3p	32	18	chr10: 133 771 747–133 773 035 (+)	protein_coding: PPP2R2D
hsa-miR-4712-3p	38	24	chr12: 50 745 528–50 747 302 (−)	protein_coding: FAM186A
hsa-miR-433-5p	44	30	chr16: 600 512–601 669 (−)	antisense: LA16c-366D1.3
hsa-miR-649	30	18	chr8: 142 262 432–142 264 762 (−)	circRNA: hsa_circ_0001829
hsa-miR-219b-5p	38	27	chr22: 21 537 410–21 538 848 (+)	processed_transcript: FAM230B
hsa-miR-6761-5p	49	39	chr14: 107 147 088–107 148 903 (−)	circRNA: hsa_circ_0033997
hsa-miR-649	28	18	chr9: 139 996 161–139 997 435 (+)	circRNA: hsa_circ_0089635
hsa-miR-4668-3p	27	17	chrX: 139 865 277–139 866 621 (−)	protein_coding: CDR1
hsa-miR-5692b	20	10	chr3: 195 607 265–195 609 187 (−)	circRNA: hsa_circ_0001377
hsa-miR-34a-3p	31	21	chr10: 133 770 676–133 771 926 (+)	protein_coding: PPP2R2D

### Web interface

**Fig 5:**
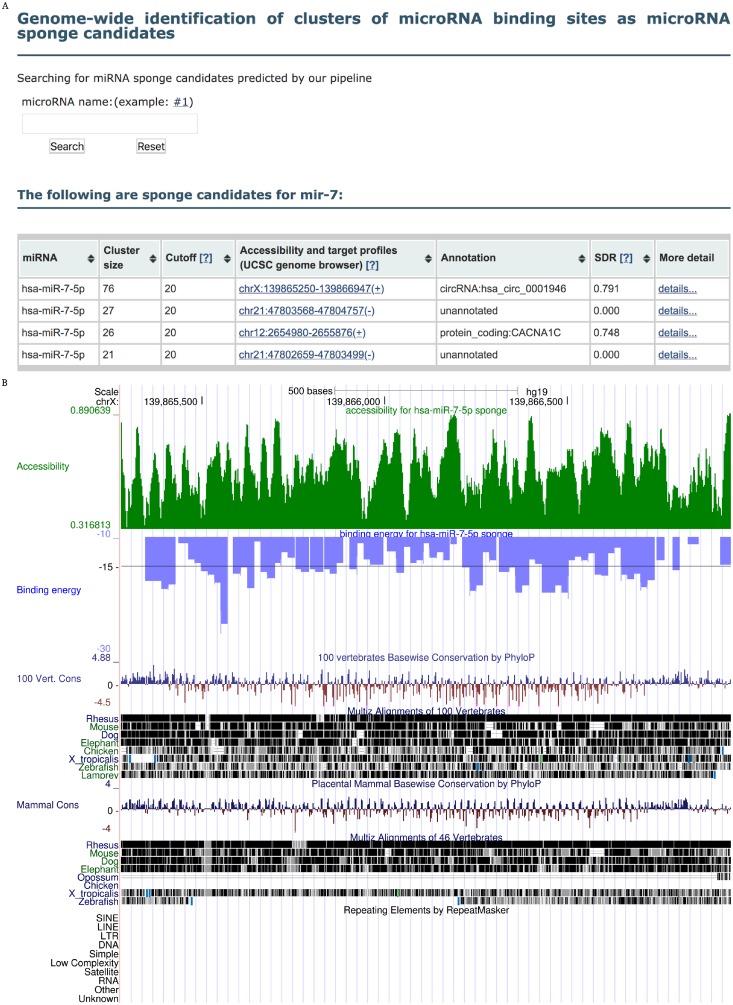
Web resource of miRNA sponge candidates. To illustrate the web resource, we show the results for miR-7. (A) When searching for a miRNA, the user is presented with an overview table of the corresponding miRNA sponge candidates. In case of miR-7, our pipeline suggests four sponge candidates, the top scoring of which is the known sponge ciRS-7 (named hsa_circ_0001946 in circBase). Clicking detail opens a page with detailed properties of this sponge candidate. (B) Clicking the coordinate of a sponge opens the UCSC genome browser with tracks showing conservation, accessibility profile (probability of the bases being unpaired in the RNA structure), and target profile (binding energies for the miRNA as predicted by RIsearch2). In the example, we show the region chrX: 139 865 280–139 866 947 (+), which corresponds to ciRS-7.

The predicted sponge candidates are freely available through a web interface at http://rth.dk/resources/mirnasponge. The interface provides the ability to search for sponge candidate for a particular miRNA of interest as well as to download the full set of sponge candidates for all miRNAs. An example for miR-7 is shown in Fig 5A. For each sponge candidate, we provide detailed information related to properties of natural miRNA sponges to assist in prioritization, including alignment details from RIsearch2, FBSE, SDR, and accessibility and target profiles with links to the UCSC genome browser (e.g. Fig 5B for miR-7 sponge ciRS-7). The help page provides a detailed explanation of all these properties.

## Discussion

Natural miRNA sponges have recently attracted much attention due to the discovery of increasing numbers of novel miRNA sponges, some of them have been linked to diseases [24, 52]. Experimental verification is still highly time-consuming and prohibitively expensive to perform systematically on a genome-wide scale. For this reason databases such as starBase v2.0 and lnCeDB rely on computational predictions of miRNA sponges; however, because the methods employed focus purely on annotated genes, they are unable to identify other genomic regions that may function as miRNA sponges.

Our study is based on the hypothesis that groups of adjacent binding sites may function together as miRNA sponges. This is consistent with a recent study showing that the number of miRNA binding sites within a sponge correlates with its ability to derepress targets of the miRNA *in vivo* [11]. We have made a genome-wide computational screen that detected potential sponge candidates through clustering of nearby miRNA binding sites. In total, we identified 3673 sponge candidates spanning 1250 miRNAs. The genome-wide analysis was made possible by the RNA–RNA interaction prediction tool RIsearch2, which can predict miRNA binding sites with high speed and with accuracy comparable to that of other methods. Although we compare cluster sizes between real and shuffled genomes and select candidates based on the differences in sizes, further refinement involves calculating p-values over all cluster sizes. However, this will require comparison across clusters based on different miRNAs as some due to their composition might have different clusters sizes than others. To the best of our knowledge, this is the first genome-wide computational approach to predict sponge candidates specifically based on high binding site density in genomic regions.

There are, however, still many limitations to prediction of miRNA sponges. Like all tools for predicting miRNA binding sites, RIsearch2 produces many true and false predictions. The latter can give rise to false predictions of miRNA sponges if they appear clustered in the genome, although our filtering steps do much to alleviate this problem.

In conclusion, we have presented a computational pipeline for discovery of clusters of putative miRNA binding sites. Interestingly, we observe an enrichment (∼2.5-fold) of clusters in protein-coding sequence which is not also annotated as circular RNA. For clusters overlapping sequence annotated both circular RNA and protein-coding sequence we observed an even stronger enrichment (∼4-fold). Both competing endogenous RNA (mRNA) and circular RNA have previously been reported to compete for miRNA binding. Hence, we consider our clusters of miRNA binding sites as miRNA sponge candidates and we in particular obtain intriguing candidates overlapping known genes.

## Supporting information

S1 Fig Overall cluster size distributions of predicted binding sites using different inflation factors. The cluster size distributions shown for both real and shuffled genomes were obtained by pooling results for all 2578 mature human miRNAs. For each miRNA, we used RIsearch2 to predict binding sites and clustered them using MCL with different inflation factors (1.5, 2.0, 2.5, 3.0, 3.5 and 4.0).
(EPS)

S2 Fig The fitting on cluster size distribution from shuffled genome for miRNAs with different GC contents. The linear function was fitted using the top-10% largest clusters obtained for the miRNA in question on the shuffled genome, where the count was transformed using log_10_. The GC content of miRNAs in A-H are 0.22, 0.35, 0.41, 0.50, 0.60, 0.71, 0.78 and 0.89, respectively.
(EPS)

S1 Table Discovered verified miRNA sponges. The table shows detailed information about the sponges, including their number of miRNA binding sites from literature and from RIsearch2 predictions.
(PDF)

S2 Table Estimated cutoff for significant clusters of 2578 miRNAs. For each miRNA, the number of clusters obtained on the shuffled genomes are fitted as linear functions of cluster size, where the counts are transformed using log_10_. In each line, it gives the fitted parameters and cutoff for significant clusters of each miRNA, and the cutoff is estimated using the fitted parameters by extrapolating the largest cluster one would expect to observe in 1000 randomizations.
(TXT)

## References

[1] Bartel DP. MicroRNAs: genomics, biogenesis, mechanism, and function. Cell. 2004;116(2):281–297. 10.1016/S0092-8674(04)00045-5 14744438

[2] Lim LP, Lau NC, Garrett-Engele P, Grimson A, Schelter JM, Castle J, et al Microarray analysis shows that some microRNAs downregulate large numbers of target mRNAs. Nature. 2005;116(2):769–773. 10.1038/nature03315 15685193

[3] Bartel DP. MicroRNAs: target recognition and regulatory functions. Cell. 2009;136(2):215–233. 10.1016/j.cell.2009.01.002 19167326 PMC3794896

[4] Gangaraju VK, Lin H. MicroRNAs: key regulators of stem cells. Nat Rev Mol Cell Biol. 2009;10(2):116–125. 10.1038/nrm2621 19165214 PMC4118578

[5] Kozomara A, Griffiths-Jones S. miRBase: integrating microRNA annotation and deep-sequencing data. Nucleic Acids Res. 2011;39:D152–157. 10.1093/nar/gkq1027 21037258 PMC3013655

[6] Winter J, Jung S, Keller S, Gregory RI, Diederichs S. Many roads to maturity: microRNA biogenesis pathways and their regulation. Nat Cell Biol. 2009;11(3):228–234. 10.1038/ncb0309-228 19255566

[7] Friedman RC, Farh KKH, Burge CB, Bartel DP. Most mammalian mRNAs are conserved targets of microRNAs. Genome Res. 2009;19(1):92–105. 10.1101/gr.082701.108 18955434 PMC2612969

[8] Esteller M. Non-coding RNAs in human disease. Nat Rev Genet. 2011;12(12):861–874. 10.1038/nrg3074 22094949

[9] Mørk S, Pletscher-Frankild S, Caro AP, Gorodkin J, Jensen LJ. Protein-driven inference of miRNA–disease associations. Bioinformatics. 2013;30(3):392–397. 10.1093/bioinformatics/btt677 24273243 PMC3904518

[10] Holohan KN, Lahiri DK, Schneider BP, Foroud T, Saykin AJ. Functional microRNAs in Alzheimer’s disease and cancer: differential regulation of common mechanisms and pathways. Front Genet. 2013;3:323 10.3389/fgene.2012.00323 23335942 PMC3547332

[11] Denzler R, Agarwal V, Stefano J, Bartel DP, Stoffel M. Assessing the ceRNA hypothesis with quantitative measurements of miRNA and target abundance. Mol Cell. 2014;54(5):766–776. 10.1016/j.molcel.2014.03.045 24793693 PMC4267251

[12] Ebert MS, Neilson JR, Sharp PA. MicroRNA sponges: competitive inhibitors of small RNAs in mammalian cells. Nat Methods. 2007;4(9):721–726. 10.1038/nmeth1079 17694064 PMC3857099

[13] Loya CM, Lu CS, Vactor DV, Fulga TA. Transgenic microRNA inhibition with spatiotemporal specificity in intact organisms. Nat Methods. 2009;6(12):897–903. 10.1038/nmeth.1402 19915559 PMC3183579

[14] Ebert MS, Sharp PA. MicroRNA sponges: progress and possibilities. RNA. 2010;16(11):2043–2050. 10.1261/rna.2414110 20855538 PMC2957044

[15] Kluiver J, Gibcus JH, Hettinga C, Adema A, Richter MKS, Halsema N, et al Rapid generation of microRNA sponges for microRNA inhibition. PloS One. 2012;7(1):e29275 10.1371/journal.pone.0029275 22238599 PMC3253070

[16] Ling H, Fabbri M, Calin GA. MicroRNAs and other non-coding RNAs as targets for anticancer drug development. Nat Rev Drug Discov. 2013;12(11):847–865. 10.1038/nrd4140 24172333 PMC4548803

[17] Ebert MS, Sharp PA. Emerging roles for natural microRNA sponges. Curr Biol. 2010;20(19):R858–R861. 10.1016/j.cub.2010.08.052 20937476 PMC4070712

[18] Salmena L, Poliseno L, Tay Y, Kats L, Pandolfi PP. A ceRNA hypothesis: the Rosetta Stone of a hidden RNA language? Cell. 2011;146(3):353–358. 10.1016/j.cell.2011.07.014 21802130 PMC3235919

[19] Hansen TB, Jensen TI, Clausen BH, Bramsen JB, Finsen B, Damgaard CK, et al Natural RNA circles function as efficient microRNA sponges. Nature. 2013;495(7441):384–388. 10.1038/nature11993 23446346

[20] Memczak S, Jens M, Elefsinioti A, Torti F, Janna Krueger AR, Maier L, et al Circular RNAs are a large class of animal RNAs with regulatory potency. Nature. 2013;495(7441):333–338. 10.1038/nature11928 23446348

[21] Hansen TB, Kjems J, Damgaard CK. Circular RNA and miR-7 in cancer. Cancer Res. 2013;73(18):5609–5612. 10.1158/0008-5472.CAN-13-1568 24014594

[22] Guo JU, Agarwal V, Guo H, Bartel DP. Expanded identification and characterization of mammalian circular RNAs. Genome Biol. 2014;15(7):1 10.1186/s13059-014-0409-z PMC4165365 25070500

[23] Zhang XO, Dong R, Zhang Y, Zhang JL, Luo Z, Zhang J, et al Diverse alternative back-splicing and alternative splicing landscape of circular RNAs. Genome Res. 2016;26(9):1277–1287. 10.1101/gr.202895.115 27365365 PMC5052039

[24] Tay Y, Kats L, Salmena L, Weiss D, Tan SM, Ala U, et al Coding-independent regulation of the tumor suppressor PTEN by competing endogenous mRNAs. Cell. 2011;147(2):344–357. 10.1016/j.cell.2011.09.029 22000013 PMC3235920

[25] Kallen AN, Zhou XB, Xu J, Qiao C, Ma J, Yan L, et al The imprinted H19 lncRNA antagonizes let-7 microRNAs. Mol Cell. 2013;52(1):101–112. 10.1016/j.molcel.2013.08.027 24055342 PMC3843377

[26] Wang Y, Xu Z, Jiang J, Xu C, Kang J, Xiao L, et al Endogenous miRNA sponge lincRNA-RoR regulates Oct4, Nanog, and Sox2 in human embryonic stem cell self-renewal. Dev Cell. 2013;25(1):69–80. 10.1016/j.devcel.2013.03.002 23541921

[27] Ee-chun C, Lin H. Repressing the repressor: a lincRNA as a MicroRNA sponge in embryonic stem cell self-renewal. Dev Cell. 2013;25(1):1–2. 10.1016/j.devcel.2013.03.020 23597480 PMC3906851

[28] Thomson DW, Dinger ME. Endogenous microRNA sponges: evidence and controversy. Nat Rev Genet. 2016 5;17(5):272–283. 10.1038/nrg.2016.20 27040487

[29] Sarver AL, Subramanian S. Competing endogenous RNA database. Bioinformation. 2012;8(15):731–733. 10.6026/97320630008731 23055620 PMC3449376

[30] Das S, Ghosal S, Sen R, Chakrabarti J. lnCeDB: Database of Human Long Noncoding RNA Acting as Competing Endogenous RNA. PloS One. 2014;9(6):e98965 10.1371/journal.pone.0098965 24926662 PMC4057149

[31] Li JH, Liu S, Zhou H, Qu LH, Yang JH. starBase v2.0: decoding miRNA-ceRNA, miRNA-ncRNA and protein–RNA interaction networks from large-scale CLIP-Seq data. Nucleic Acids Res. 2014;42:D92–D97. 10.1093/nar/gkt1248 24297251 PMC3964941

[32] Le TD, Zhang J, Liu L, Li J. Computational methods for identifying miRNA sponge interactions. Brief Bioinform. 2016;p. bbw042. 10.1093/bib/bbw042 27273287

[33] Rosenbloom KR, Armstrong J, Barber GP, Casper J, Clawson H, Diekhans M, et al The UCSC Genome Browser database: 2015 update. Nucleic Acids Res. 2015 1;43(Database issue):D670–D681. 10.1093/nar/gku1177 25428374 PMC4383971

[34] Harrow J, Frankish A, Gonzalez JM, Tapanari E, Diekhans M, Kokocinski F, et al GENCODE: the reference human genome annotation for The ENCODE Project. Genome Res. 2012;22(9):1760–1774. 10.1101/gr.135350.111 22955987 PMC3431492

[35] Glažar P, Papavasileiou P, Rajewsky N. circBase: a database for circular RNAs. RNA. 2014;20(11):1666–1670. 10.1261/rna.043687.113 25234927 PMC4201819

[36] Pollard KS, Hubisz MJ, Rosenbloom KR, Siepel A. Detection of nonneutral substitution rates on mammalian phylogenies. Genome Res. 2010;20(1):110–121. 10.1101/gr.097857.109 19858363 PMC2798823

[37] Alkan F, Wenzel A, Palasca O, Kerpedjiev P, Rudebeck AF, Stadler PF, et al RIsearch2: suffix array-based large-scale prediction of RNA–RNA interactions and siRNA off-targets. Nucleic Acids Res. 2017;Forthcoming. 10.1093/nar/gkw1325 28108657 PMC5416843

[38] Wenzel A, Akbaşlı E, Gorodkin J. RIsearch: fast RNA–RNA interaction search using a simplified nearest-neighbor energy model. Bioinformatics. 2012;28(21):2738–2746. 10.1093/bioinformatics/bts519 22923300 PMC3476332

[39] van Dongen S. Graph clustering by flow simulation [PhD dissertation]. University of Utrecht; 2000. Available from: http://dspace.library.uu.nl/handle/1874/848.

[40] Jiang M, Anderson J, Gillespie J, Mayne M. uShuffle: a useful tool for shuffling biological sequences while preserving the k-let counts. BMC Bioinformatics. 2008;9(1):1 10.1186/1471-2105-9-192 18405375 PMC2375906

[41] Anthon C, Tafer H, Havgaard JH, Thomsen B, Hedegaard J, Seemann SE, et al Structured RNAs and synteny regions in the pig genome. BMC Genomics. 2014;15(1):1 10.1186/1471-2164-15-459 24917120 PMC4124155

[42] Lange SJ, Maticzka D, Möhl M, Gagnon JN, Brown CM, Backofen R. Global or local? Predicting secondary structure and accessibility in mRNAs. Nucleic Acids Res. 2012;40(12):5215–5226. 10.1093/nar/gks181 22373926 PMC3384308

[43] Kohany O, Gentles AJ, Hankus L, Jurka J. Annotation, submission and screening of repetitive elements in Repbase: RepbaseSubmitter and Censor. BMC Bioinformatics. 2006;7:7:474 10.1186/1471-2105-7-474 17064419 PMC1634758

[44] Smit AF, Hubley R, Green P. RepeatMasker Open-3.0; 1996. Available from: http://www.repeatmasker.org.

[45] Thomas LF, Sætrom P. Circular RNAs are depleted of polymorphisms at microRNA binding sites. Bioinformatics. 2014;30(16):2243–2246. 10.1093/bioinformatics/btu257 24764460 PMC4207428

[46] Cunningham F, Amode MR, Barrell D, Beal K, Billis K, Brent S, et al Ensembl 2015. Nucleic Acids Res. 2015;43(D1):D662–D669. 10.1093/nar/gku1010 25352552 PMC4383879

[47] Junge A, Garde C, Refsgaard JC, Pan X, Santos A, Alkan F, et al RAIN: RNA–protein Association and Interaction Networks. Database. 2017;2017(1):baw167 10.1093/database/baw167 PMC5225963 28077569

[48] Bernhart SH, Mückstein U, Hofacker IL. RNA Accessibility in cubic time. Algorithms Mol Biol. 2011;6(1):1 10.1186/1748-7188-6-3 21388531 PMC3063221

[49] Gerlach W, Giegerich R. GUUGle: a utility for fast exact matching under RNA complementary rules including G–U base pairing. Bioinformatics. 2006;22(6):762–764. 10.1093/bioinformatics/btk041 16403789

[50] Altschul SF, Madden TL, Schäffer AA, Zhang J, Zhang Z, Miller W, et al Gapped BLAST and PSI-BLAST: a new generation of protein database search programs. Nucleic Acids Res. 1997;25(17):3389–3402. 10.1093/nar/25.17.3389 9254694 PMC146917

[51] Lewis BP, Burge CB, Bartel DP. Conserved Seed Pairing, Often Flanked by Adenosines, Indicates that Thousands of Human Genes are MicroRNA Targets. Cell. 2005;120(1):15–20. 10.1016/j.cell.2004.12.035 15652477

[52] Liu XH, Sun M, Nie FQ, Ge YB, Zhang EB, Yin DD, et al LncRNA HOTAIR functions as a competing endogenous RNA to regulate HER2 expression by sponging miR-331-3p in gastric cancer. Mol Cancer. 2014;13(1):1 10.1186/1476-4598-13-92 24775712 PMC4021402

