# Supplementary material for: Genome-wide identification of clusters of predicted microRNA binding sites as microRNA sponge candidates
Source: PLoS One. 2018 Aug 24;13(8):e0202369. doi: 10.1371/journal.pone.0202369 (PMC6108476; doi:10.1371/journal.pone.0202369)
Supplement: S1 Table — The table shows detailed information about the sponges, including their number of miRNA binding sites from literature and from RIsearch2 predictions. (PDF) [file pone.0202369.s003.pdf]

Discovered natural miRNA sponges and ceRNAs

In Table 1 we provide a list of validated endogeneous miRNA sponges extracted from the literature. We always use the term 'sponge' here, also for protein-coding RNAs that share miRNA binding sites with other mRNAs in their UTRs, and are more commonly referred to as ceRNA. We searched PubMed and Google Scholar with key words 'miRNA/microRNA/miR sponge' and 'competing endogenous RNA/miRNA ceRNA' on 06/2014. For 'miRNA/microRNA/miR sponge' we found about 200 publications for 'competing endogenous RNA/miRNA ceRNA' we found 120 papers. We manually looked through the articles, and collected information on validated sponges. We also looked into the relevant references, especially for reviews, such as the one by Tay et al. [1]. If there were no coordinates given in the original papers, coordinates for non-coding RNAs, such as lncRNAs or pseudogenes, were retrieved from lncRNAdb; for mRNAs coordinates, including coding region and 3' UTR, were extracted from UCSC browser.

S1 Table. Discovered verified miRNA sponges. The table gives detailed information about sponges found from the literature, coordinates refer to hg19 or mm9 respectively. For the number of miRNA binding sites from literature it should be noted that they mostly are based on predictions as well. If the number of sites is not stated in the reference, we note '-'. For RIsearch2, we used the same settings as described for the analysis in the main paper and run against entire target sequence instead of only 3' UTR.

Sponge	Organism	Coordinate	miRNA	# binding sites from literature	# binding sites from RIsearch2	Reference
CDR1as/ciRS-7	Human	chrX: 139 865 328–139 866 829 (+)	miR-7	73	73	[2, 3]
OCT4-pg4	Human	chr1: 155 402 971–155 403 534 (+)	miR-145	2	3	[4]
lincRNA-RoR	Human	chr18: 54 721 802–54 739 350 (−)	miR-145	2	22	[5]
H19	Human	chr11: 2 016 406–2 019 065 (−)	let-7 family	-	13	[6]
HULC	Human	chr6: 8 652 369–8 654 079 (+)	miR-372	1	2	[7]
loc285194	Human	chr3: 116 428 625–116 442 428 (+)	miR-211	2	28	[8]
PTCSC3	Human	chr14: 36 604 916–36 645 857 (−)	miR-574	2	39	[9]
HOTAIR	Human	chr12: 54 356 092–54 368 740 (−)	miR-331	11	55	[10]
CARL	Human	chr10: 22 764 252–22 766 310 (−)	miR-539	1	2	[11]
PTENP1	Human	chr9: 33 673 502–33 677 418 (−)	miR-26	1	5	[12]
			miR-17	1	1	
			miR-19	1	1	
			miR-21	1	2	
			miR-214	1	4	
TUSC2P	Human	chrY: 5 887 511–5 888 741 (+)	miR-608	4	6	[13]
			miR-17	1	2	
			miR-661	2	3	
			miR-299-3p	1	2	
			miR-93	1	2	
			miR-520a	1	2	
KRAS1P	Human	chr6: 54 635 169–54 636 037 (+)	let-7 family	8(3)	1	[12]
			miR-143	2	0	
CNOT6L	Human	chr4: 78 634 541–78 740 522 (−)	miR-17	-	82	[14]
			miR-19 family	-	37	
VAPA	Human	chr18: 9 913 955–9 960 018 (+)	miR-17	-	35	[14]
			miR-19 family	-	23	
			miR-26 family	-	25	
VCAN	Human	chr5: 82 767 493–82 808 758 (+)	miR-144	1	10	[15]
			miR-136	1	33	
			miR-199a	1	30	
CD34	Human	chr1: 208 059 883–208 084 683 (−)	miR-133a	1	68	[16]
			miR-144	1	5	
			miR-431	2	73	
FN1	Human	chr2: 216 225 179–216 257 926 (−)	miR-133a	1	43	[16, 17]
			miR-199a	1	40	
			miR-431	1	89	
CD44	Human	chr11: 35 160 417–35 244 867 (+)	miR-491	-	340	[18]
			miR-512	-	191	
			miR-671	-	307	
			miR-216a	-	77	[19]
			miR-330	-	88	
			miR-608	-	754	
ZEB2	Human	chr2: 145 141 942–145 277 958 (−)	miR-25	1	351	[20]
			miR-181	1	110	
			miR-200b	6	215	
			miR-92a	1	49	
Sry	Mouse	chrY: 1 918 440–1 919 671 (−)	miR-138	16	21	[2]
linc-MD1	Mouse	chr1: 20 659 963–20 673 039 (−)	miR-133a	1	5	[21]
			miR-133b	1	7	
			miR-135a	1	3	
			miR-135b	2	3	
CHRF	Mouse	chr18: 72 164 057–72 165 898 (−)	miR-489	1	6	[22]

References

[1] Tay Y, Rinn J, Pandolfi PP. The multilayered complexity of ceRNA crosstalk and competition. Nature. 2014 Jan 16;505(7483):344-52. doi: 10.1038/nature12986.

[2] Hansen TB, Jensen TI, Clausen BH, Bramsen JB, Finsen B, Damgaard CK, et al. Natural RNA circles function as efficient microRNA sponges. Nature. 2013 Mar 21;495(7441):384-8. doi: 10.1038/nature11993.

[3] Memczak S, Jens M, Elefsinioti A, Torti F, Krueger J, Rybak A, et al. Circular RNAs are a large class of animal RNAs with regulatory potency. Nature. 2013 Mar 21;495(7441):333-8. doi: 10.1038/nature11928.

[4] Wang L, Guo ZY, Zhang R, Xin B, Chen R, Zhao J, Wang T, Wen WH, Jia LT, Yao LB, Yang AG. Pseudogene OCT4-pg4 functions as a natural micro RNA sponge to regulate OCT4 expression by competing for miR-145 in hepatocellular carcinoma. Carcinogenesis. 2013 Aug;34(8):1773-81. doi: 10.1093/carcin/bgt139.

[5] Wang Y, Xu Z, Jiang J, Xu C, Kang J, Xiao L, Wu M, Xiong J, Guo X, Liu H. Endogenous miRNA sponge lincRNA-RoR regulates Oct4, Nanog, and Sox2 in human embryonic stem cell self-renewal. Dev Cell. 2013 Apr 15;25(1):69-80. doi: 10.1016/j.devcel.2013.03.002.

[6] Kallen AN, Zhou XB, Xu J, Qiao C, Ma J, Yan L, Lu L, Liu C, Yi JS, Zhang H, Min W, Bennett AM, Gregory RI, Ding Y, Huang Y. The imprinted H19 lncRNA antagonizes let-7 microRNAs. Mol Cell. 2013 Oct 10;52(1):101-12. doi: 10.1016/j.molcel.2013.08.027.

[7] Wang J, Liu X, Wu H, Ni P, Gu Z, Qiao Y, Chen N, Sun F, Fan Q. CREB up-regulates long non-coding RNA, HULC expression through interaction with microRNA-372 in liver cancer. Nucleic Acids Res. 2010 Sep;38(16):5366-83. doi: 10.1093/nar/gkq285.

[8] Liu Q, Huang J, Zhou N, Zhang Z, Zhang A, Lu Z, Wu F, Mo YY. LncRNA loc285194 is a p53-regulated tumor suppressor. Nucleic Acids Res. 2013 May;41(9):4976-87. doi: 10.1093/nar/gkt182.

[9] Fan M, Li X, Jiang W, Huang Y, Li J, Wang Z. A long non-coding RNA, PTCSC3, as a tumor suppressor and a target of miRNAs in thyroid cancer cells. Exp Ther Med. 2013 Apr;5(4):1143-1146.

[10] Liu XH, Sun M, Nie FQ, Ge YB, Zhang EB, Yin DD, Kong R, Xia R, Lu KH, Li JH, De W, Wang KM, Wang ZX. Lnc RNA HOTAIR functions as a competing endogenous RNA to regulate HER2 expression by sponging miR-331-3p in gastric cancer. Mol Cancer. 2014 Apr 28;13:92. doi: 10.1186/1476-4598-13-92.

[11] Wang K, Long B, Zhou LY, Liu F, Zhou QY, Liu CY, Fan YY, Li PF. CARL lncRNA inhibits anoxia-induced mitochondrial fission and apoptosis in cardiomyocytes by impairing miR-539-dependent PHB2 downregulation. Nat Commun. 2014 Apr 7;5:3596. doi: 10.1038/ncomms4596.

[12] Poliseno L, Salmena L, Zhang J, Carver B, Haveman WJ, Pandolfi PP. A coding-independent function of gene and pseudogene mRNAs regulates tumour biology. Nature. 2010 Jun 24;465(7301):1033-8. doi: 10.1038/nature09144.

[13] Rutnam ZJ, Du WW, Yang W, Yang X, Yang BB. The pseudogene TUSC2P promotes TUSC2 function by binding multiple microRNAs. Nat Commun. 2014;5:2914. doi: 10.1038/ncomms3914.

[14] Tay Y, Kats L, Salmena L, Weiss D, Tan SM, Ala U, Karreth F, Poliseno L, Provero P, Di Cunto F, Lieberman J, Rigoutsos I, Pandolfi PP. Coding-independent regulation of the tumor suppressor PTEN by competing endogenous mRNAs. Cell. 2011 Oct 14;147(2):344-57. doi: 10.1016/j.cell.2011.09.029.

[15] Lee DY, Jeyapalan Z, Fang L, Yang J, Zhang Y, Yee AY, Li M, Du WW, Shatseva T, Yang BB. Expression of versican 3'-untranslated region modulates endogenous microRNA functions. PLoS One. 2010 Oct 25;5(10):e13599. doi: 10.1371/journal.pone.0013599.

[16] Fang L, Du WW, Yang X, Chen K, Ghanekar A, Levy G, Yang W, Yee AJ, Lu WY, Xuan JW, Gao Z, Xie F, He C, Deng Z, Yang BB. Versican 3'-untranslated region (3'-UTR) functions as a ceRNA in inducing the development of hepatocellular carcinoma by regulating miRNA activity. FASEB J. 2013 Mar;27(3):907-19. doi: 10.1096/fj.12-220905.

[17] Lee DY, Shatseva T, Jeyapalan Z, Du WW, Deng Z, Yang BB. A 3'-untranslated region (3'UTR) induces organ adhesion by regulating miR-199a* functions. PLoS One. 2009;4(2):e4527. doi: 10.1371/journal.pone.0004527.

[18] Rutnam ZJ, Yang BB. The non-coding 3' UTR of CD44 induces metastasis by regulating extracellular matrix functions. J Cell Sci. 2012 Apr 15;125(Pt 8):2075-85. doi: 10.1242/jcs100818.

[19] Jeyapalan Z, Deng Z, Shatseva T, Fang L, He C, Yang BB. Expression of CD44 3'-untranslated region regulates endogenous microRNA functions in tumorigenesis and angiogenesis. Nucleic Acids Res. 2011 Apr;39(8):3026-41. doi: 10.1093/nar/gkq1003.

[20] Karreth FA, Tay Y, Perna D, Ala U, Tan SM et al., In vivo identification of tumor- suppressive PTEN ceRNAs in an oncogenic BRAF-induced mouse model of melanoma. Cell. 2011 Oct 14;147(2):382-95. doi: 10.1016/j.cell.2011.09.032.

[21] Cesana M, Cacchiarelli D, Legnini I, Santini T, Sthandier O, Chinappi M, Tramontano A, Bozzoni I. A long noncoding RNA controls muscle differentiation by functioning as a competing endogenous RNA. Cell. 2011 Oct 14;147(2):358-69. doi: 10.1016/j.cell.2011.09.028.

[22] Wang K, Liu F, Zhou LY, Long B, Yuan SM, Wang Y, Liu CY, Sun T, Zhang XJ, Li PF. The long noncoding RNA CHRF regulates cardiac hypertrophy by targeting miR-489. Circ Res. 2014 Apr 25;114(9):1377-88. doi: 10.1161/CIRCRESAHA.114.302476.
